# Na^+^/K^+^-ATPase α1 mRNA expression in the gill and rectal gland of the Atlantic stingray, *Dasyatis sabina*, following acclimation to increased salinity

**DOI:** 10.1186/s13104-015-1216-7

**Published:** 2015-06-05

**Authors:** Andrew N Evans, Faith N Lambert

**Affiliations:** Department of Coastal Sciences, Gulf Coast Research Laboratory, University of Southern Mississippi, 703 East Beach Drive, Ocean Springs, MS USA

**Keywords:** Na^+^/K^+^-ATPase, Rectal gland, Osmoregulation, Elasmobranch, Euryhaline

## Abstract

**Background:**

The salt-secreting rectal gland plays a major role in elasmobranch osmoregulation, facilitating ion balance in hyperosmotic environments in a manner analogous to the teleost gill. Several studies have examined the central role of the sodium pump Na^+^/K^+^-ATPase in osmoregulatory tissues of euryhaline elasmobranch species, including regulation of Na^+^/K^+^-ATPase activity and abundance in response to salinity acclimation. However, while the transcriptional regulation of Na^+^/K^+^-ATPase in the teleost gill has been well documented the potential for mRNA regulation to facilitate rectal gland plasticity during salinity acclimation in elasmobranchs has not been examined. Therefore, in this study we acclimated Atlantic stingrays, *Dasyatis sabina* (Lesueur) from 11 to 34 ppt salinity over 3 days, and examined changes in plasma components as well as gill and rectal gland Na^+^/K^+^-ATPase α1 (*atp1a1*) mRNA expression.

**Results:**

Acclimation to increased salinity did not affect hematocrit but resulted in significant increases in plasma osmolality, chloride and urea. Rectal gland *atp1a1* mRNA expression was higher in 34 ppt-acclimated *D. sabina* vs. controls. There was no significant change in gill *atp1a1* mRNA expression, however mRNA expression of this gene in the gill and rectal gland were negatively correlated.

**Conclusions:**

This study demonstrates regulation of *atp1a1* in the elasmobranch salt-secreting gland in response to salinity acclimation and a negative relationship between rectal gland and gill *atp1a1* expression. These results support the hypothesis that the gill and rectal gland play opposing roles in ion balance with the gill potentially facilitating ion uptake in hypoosmotic environments. Future studies should further examine this possibility as well as potential differences in the regulation of Na^+^/K^+^-ATPase gene expression between euryhaline and stenohaline elasmobranch species.

## Background

Significant aspects of elasmobranch osmoregulatory physiology include a heavy reliance on nitrogenous compounds for urea-based osmoregulation and the presence of the salt-secreting rectal gland, an organ that is unique to these taxa. The rectal gland is the primary site for sodium and chloride secretion in euryhaline and marine elasmobranchs, an osmoregulatory role analogous to that of the teleost gill. Therefore, in contrast to the teleost fishes the elasmobranch gill is thought to play a comparatively lesser role in salt balance. However, a series of studies in the Atlantic stingray (*Dasyatis sabina*) demonstrated changes in gill ion exchange proteins in response to changes in environmental salinity and suggested a role for the elasmobranch gill in ion uptake in addition to acid–base regulation [1–3]. Therefore the elasmobranch gill may play a more significant role in ion balance than previously accepted, particularly in euryhaline species that may experience frequent and/or rapid changes in salinity.

The Atlantic stingray is an excellent model for studies regarding the osmoregulatory physiology of elasmobranchs, as this euryhaline species is a common inhabitant of coastal bays and estuaries ranging from the east coast of the United States to Central America [4]. *D. sabina* therefore experience daily and seasonal fluctuations in environmental salinity and have been collected from coastal areas with salinities ranging from 2.2 to 36.7 ppt [5–7] as well as in hypersaline habitats such as the Laguna Madre in south Texas [8]. Furthermore, it has been reported that some populations of Atlantic stingrays migrate into freshwater rivers [9, 10], and a permanent freshwater population is established in the St. John’s River of Florida [11].

The enzyme Na^+^/K^+^-ATPase plays a central role in ion transport and is highly abundant in secretory cells of both the gill (ionocytes) and rectal gland, driving the secondary active transport of chloride involving ion channels and symport proteins such as the Na–K–Cl cotransporter [12, 13]. Multiple studies have specifically examined the osmoregulatory role of Na^+^/K^+^-ATPase in the gill and rectal gland of euryhaline elasmobranch fishes challenged with changes in salinity. In the bull shark (*Carcharhinus leucas*), seawater acclimation increases Na^+^/K^+^-ATPase activity in rectal gland with no corresponding change in gill Na^+^/K^+^-ATPase activity [14]. Rectal gland Na^+^/K^+^-ATPase abundance and activity also increase in *D. sabina* following seawater acclimation, with a corresponding decrease in gill Na^+^/K^+^-ATPase activity along with changes in gill protein localization [1]. Finally, it has been demonstrated that gill Na^+^/K^+^-ATPase alpha subunit (*atp1a1*) mRNA expression in both *D. sabina* and *C. leucas* is higher in freshwater vs. seawater individuals, supporting the hypothesis that the elasmobranch gill plays a role in ion uptake and also suggesting that transcriptional regulation is one mechanism by which euryhaline taxa respond to salinity challenges [15, 16].

Na^+^/K^+^-ATPase activity of the elasmobranch rectal gland is more than tenfold higher than that of gill [1, 14], and this organ is critical for ion balance in euryhaline and marine elasmobranch species. It is therefore important to understand potential mechanisms for regulation of the activity of this gland, including transcriptional, translational and post-translational processes. Changes in rectal gland *atp1a1* mRNA expression following feeding in the stenohaline European dogfish (*Scyliorhinus canicula*) and spiny dogfish (*Squalus acanthias*) have been reported, supporting the hypothesis that transcriptional mechanisms play a role in the plasticity of this organ in response to salt-loading [17, 18]. However, while changes in Na^+^/K^+^-ATPase activity in response to altered salinity has been demonstrated as described above, the potential role for mRNA regulation in the rectal gland to facilitate salinity acclimation has not been examined. Therefore, for the laboratory component of the 2014 Summer Field Program undergraduate course *Stingray Physiology* at the University of Southern Mississippi Gulf Coast Research Laboratory, we examined the regulation of plasma components as well as *atp1a1* mRNA expression in the gill and rectal gland of *D. sabina* acclimated to increased environmental salinity.

## Methods

### Animals

Atlantic stingrays were captured by otter trawl in coastal waters near Ocean Springs, Mississippi (salinity ~11 ppt). A total of six mature (>22 cm disk width; [19]) animals were used for this study (two males, four females; disk width: 23–29 cm; mass: 0.5–1.0 kg). Stingrays were transferred to the laboratory and maintained at 11 ppt and ambient temperature (water temperature 26–27°C) in recirculating 1,700 L tanks. Animals were fed chopped shrimp ad libitum every other day and were acclimated to captivity for at least 2 weeks prior to experimentation. Following completion of the experiment animals were sacrificed by immersion in 1 g L^−1^ buffered tricaine methanesulfonate (MS-222) and subsequent severing of the spinal column posterior to the gill arches, as approved by the University of Southern Mississippi Animal Care and Use Committee (IACUC protocol #13031403).

### Salinity acclimation

Stingrays (two tanks, n = 3 per tank) were maintained at 11 ppt prior to the start of the salinity acclimation experiment. Before salinity acclimation, individual stingrays were removed from the tank by net and 0.5 mL blood was drawn from the wing of each animal using a 22-gauge needle on a 1 mL syringe. Blood was immediately transferred to lithium heparin vacutainers and placed on ice. After hematocrit determination using whole blood as described below, samples were centrifuged for 5 min at 5,000×*g*, with plasma transferred to a new microcentrifuge tube and stored at −80°C. Following a recovery period of 4 h, salinity acclimation was initiated by adding concentrated seawater brine (60–90 ppt) to the experimental tank’s filter box to facilitate a step-wise increase in salinity from 11 to 34 ppt over the course of 3 days (15–25–34 ppt). This protocol is similar to that used in previous SW-acclimation studies using the Atlantic stingray as a model species, in which animals were acclimated from brackish salinities (15–16 ppt) to seawater (30–32 ppt) over a period of 2–3 days [1, 20]. Stingrays were not fed during this time, and animals from both tanks were sampled 1 day after the salinity increase was completed (day four). Following blood collection as described above, individuals were sacrificed and rectal glands were then rapidly removed, coarsely minced and placed in microcentrifuge tubes containing RNA*later* (Ambion). To collect gill tissue, a single gill arch was removed from each individual and scraped using a sterile scalpel blade; all tissue was then rapidly transferred to a microcentrifuge tube containing RNA*later*.

### Plasma analyses

All plasma analyses were completed in duplicate. Plasma osmolality was determined using a Vapro 5520 vapor pressure osmometer (Wescor), and chloride was quantified using a digital chloridometer (Labconco). For determining urea concentrations, plasma was diluted 1:50 and analyzed using a commercial colorimetric assay (QuantChrom Urea Assay, Bioassay Systems). Hematocrit was measured in approximately 50 µL of whole blood per replicate using 75 mm ammonium-heparin hematocrit tubes and an IEC Micro MB microhematocrit centrifuge (Thermo), with values determined using a microhematocrit capillary tube reader disk.

### RNA isolation, cDNA synthesis and quantitative PCR

Total RNA was isolated from rectal gland and gill tissue using TRI Reagent (Zymo Research), following the manufacturer’s instructions. Five µg of total RNA from each sample was then DNase treated and recovered using a Zymo Research RNA Clean and Concentrator kit with in-column DNA digestion. One microgram of total DNase-treated RNA was then used as template for reverse transcriptase (RT) reactions using the Maxima First Strand cDNA Synthesis Kit for RT-qPCR (Thermo Scientific). SYBR green real time quantitative PCR (qRT-PCR) was used to quantify relative levels of *atp1a1* mRNA using primers previously described for *D. sabina* [15]. For qRT-PCR, 2 µL of each RT reaction was added to a 20 µL total volume reaction containing 10 µL SYBR Select 2× master mix (Applied Biosystems) and 250 nM each gene-specific sense and antisense primers (Table 1). qRT-PCR reactions were cycled in 96-well plates using an Applied Biosystems 7500 Fast Real-Time PCR System with the following parameters: 50°C for 2 min then 95°C for 2 min, followed by 40 cycles of 95°C for 15 s and 60°C for 1 min. Dissociation curves were determined to verify that a single product was produced in each reaction, and no-template controls were included in each run. All reactions were run in triplicate, with *atp1a1* mRNA levels normalized to *18S* rRNA levels determined in separate qRT-PCR reactions and relative mRNA levels determined using the equation described by Fink et al. [21]. The expression of *18S* was unaffected by salinity acclimation in both tissues, and *atp1a1* and *18S* qRT-PCR amplification efficiencies were determined to be 89 and 100%, respectively.

**Table 1:** qRT-PCR primer sets and reaction efficiencies

Gene	Primer/probe	Sequence	Efficiency (%)
*18S*	Forward primer	5′-GTTAATTCCGATAACGAACGAGACTC-3′	100
	Reverse primer	5′-ACAGACCTGTTATTGCTCAATCTCGTG-3′	
*atp1a1* ^a^	Forward primer	5′-CTCTCACTCATCTTGGGATACAG-3′	89
	Reverse primer	5′-GGCATCTCCAGCAACACTT-3′	

^a^Choe et al. [15].

### Statistical analyses

Paired Student’s *t* test comparisons were used for statistical analysis of pre- and post-acclimation plasma component values within experimental groups. Rectal gland and gill *atp1a1* mRNA levels were compared using ANOVA followed by a Student–Newman–Keuls post hoc test. In all cases, p-values less than 0.05 were considered significant.

## Results and discussion

**Figure 1:**
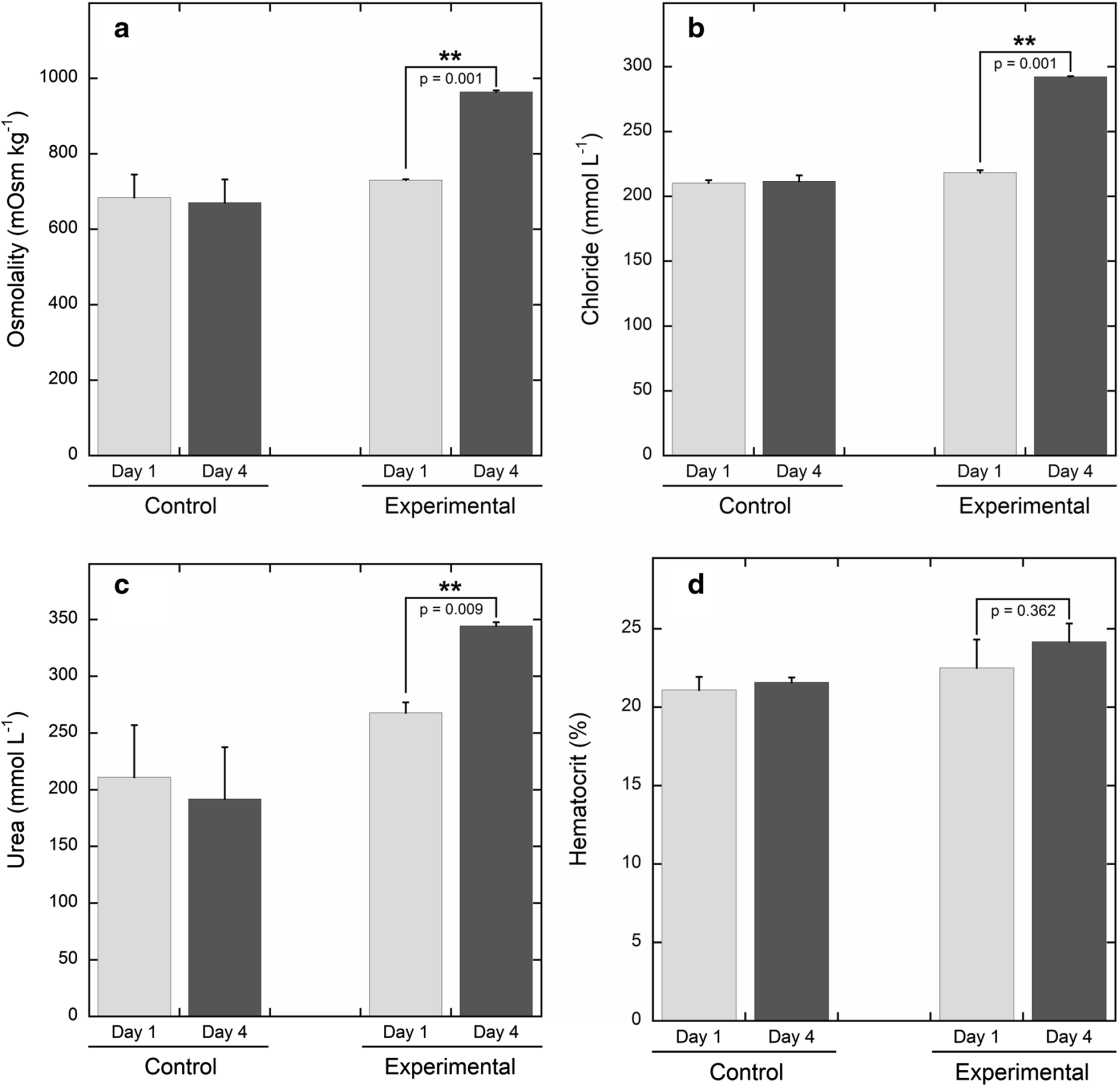
Plasma components and salinity acclimation. Day one and day four plasma osmolality (**a**), chloride (**b**), urea (**c**) and whole blood hematocrit (**d**) of control *D. sabina* vs. animals challenged with a salinity increase from 11 to 34 ppt. *Asterisks* indicate significant differences between day one and day four values within groups.

In this study we demonstrate for the first time the regulation of *atp1a1* mRNA expression in the elasmobranch rectal gland in response to salinity acclimation, supporting the hypothesis that transcriptional regulation plays a role in the osmoregulatory plasticity of the euryhaline Atlantic stingray, *Dasyatis sabina*. Acclimation of *D. sabina* from 11 to 34 ppt over a three-day period resulted in significant increases in plasma osmolality, chloride and urea (Figure 1). However, there was no significant difference between pre- and post-acclimation hematocrit, which is indicative of efficient osmoregulatory function. Further, in the control group there were no significant changes in any measured plasma component (osmolality, chloride, urea, hematocrit; Figure 1). The observed changes in plasma osmolality, chloride and urea concentrations in response to increased salinity are consistent with the osmoregulatory strategy of euryhaline elasmobranchs and have been described previously for this species [20, 22]. Our data from SW-acclimated animals are comparable to those reported by Piermarini and Evans (1998) for marine *D. sabina*, with very similar plasma chloride (292.2 [this study] vs. 300.0 [20] mmol L^−1^) but somewhat lower total osmolality (963.8 vs. 1034 mOsm kg^−1^). This gap in total osmolality is most likely due to lower plasma urea (344.5 mmol L^−1^) in SW-acclimated stingrays, which were exposed to seawater for a short period of time (24 h) compared to long-term marine *D. sabina* (394.5 mmol L^−1^ [20]). Therefore lower urea concentrations in the current study may represent ongoing changes in the rate of urea synthesis and retention in response to increased environmental salinity. A similar delay is observed in elasmobranchs acclimating to decreased salinities, in which reported urea concentrations are dependent not only on salinity but also the duration of exposure with longer exposures to hypoosmotic conditions resulting in lower urea levels [23].

**Figure 2:**
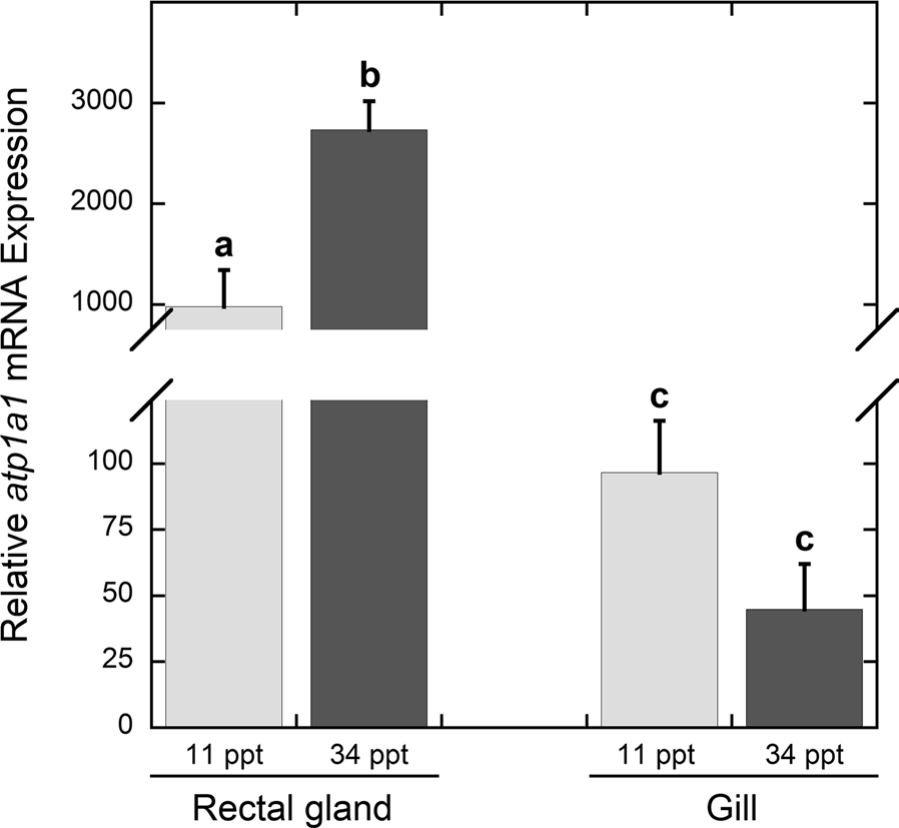
Na^+^/K^+^-ATPase α1 mRNA expression. Relative Na^+^/K^+^-ATPase α1 (*atp1a1*) mRNA expression in the gill and rectal gland of control *D. sabina* vs. animals challenged with a salinity increase from 11 to 34 ppt. Means with *different letters* are significantly different (p < 0.05).

**Figure 3:**
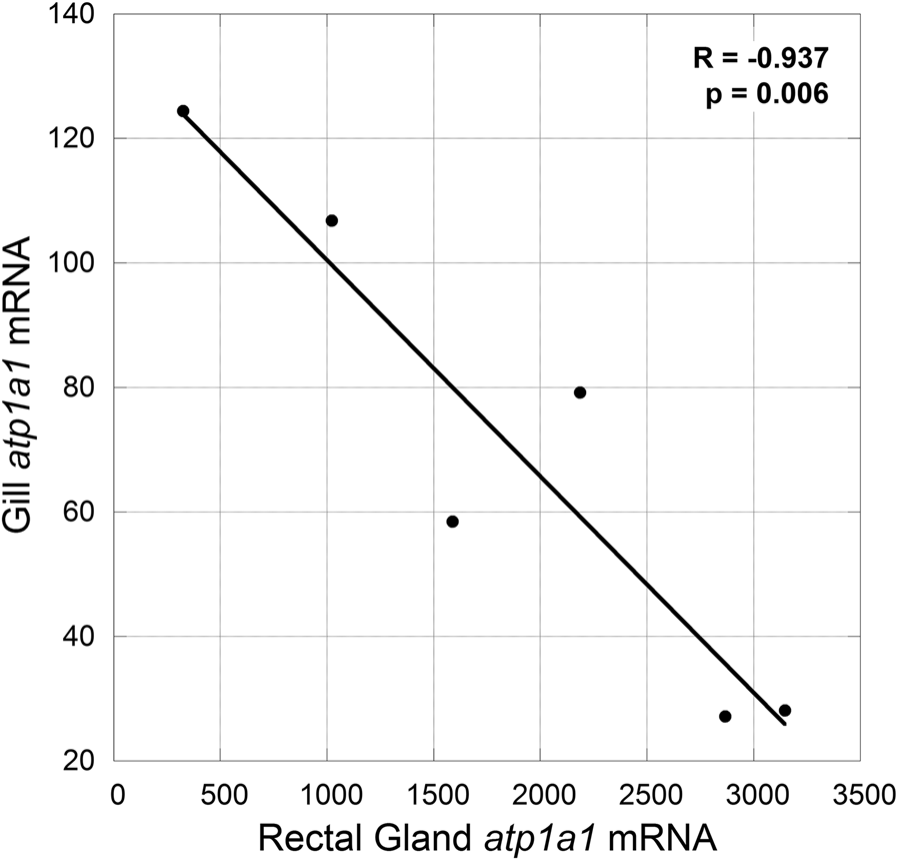
Gill vs. rectal gland Na^+^/K^+^-ATPase α1 mRNA expression. Linear regression of gill vs. rectal gland Na^+^/K^+^-ATPase α1 (*atp1a1*) mRNA expression, including both control and experimental animals (n = 6).

With regards to the regulation of Na^+^/K^+^-ATPase-driven secondary active transport of ions, previous studies have demonstrated significant changes in Na^+^/K^+^-ATPase activity in euryhaline elasmobranch species including *D. sabina* and the bull shark, *Carcharhinus leucas*, following seawater acclimation [1, 14]. Altered Na^+^/K^+^-ATPase activity has also been reported for stenohaline species such as the marine South American skate (*Zapteryx brevirostris*), in which decreased salinity does not affect gill Na^+^/K^+^-ATPase but results in lower enzyme activity in the rectal gland [23]. It is likely that changes in enzyme activity occur at several levels, including the potential activation of latent Na^+^/K^+^-ATPase protein or translation of steady-state mRNAs, which would facilitate rapid plasticity of the elasmobranch gill and rectal gland in response to frequent changes in environmental salinity. Here we show that short-term acclimation to increased salinity is accompanied by elevated *atp1a1* mRNA in the rectal gland of *D. sabina* (p = 0.019; Figure 2), supporting a role for changes in gene transcription in facilitating salinity acclimation. It may therefore be expected that acclimation of euryhaline elasmobranch species to decreased salinity is facilitated in part by a decrease in rectal gland *atp1a1* mRNA expression, and future studies should examine this possibility. Similar changes in Na^+^/K^+^-ATPase mRNA expression in response to salinity acclimation have been observed in the teleost gill, the analogous salt secreting tissue in these taxa (e.g. [24, 25], and [26]: meta-analysis of 59 studies including other taxa). With regards to Na^+^/K^+^-ATPase mRNA expression in the elasmobranch gill, in the euryhaline *D. sabina* and *C. leucas*, increased expression of gill Na^+^/K^+^-ATPase mRNA was observed in fresh water vs. seawater [15, 16]. Interestingly, expression of gill Na^+^/K^+^-ATPase α3 mRNA increases in response to brackish water (20 ppt) acclimation in the freshwater white-rimmed stingray (*Himantura signifer*), suggesting that the gill may facilitate ion secretion in freshwater species that lack a rectal gland [27]. The lack of a significant change in gill *atp1a1* mRNA expression in the current study (p = 0.119; Figure 2) may be explained by the hypothesized minor or non-existent role for this tissue in salt secretion, as supported by the lower abundance of *atp1a1* mRNA as well as previously reported differences in Na^+^/K^+^-ATPase activity in gill vs. rectal gland [1, 14]. It is also possible that we were unable to detect a significant difference in gill *atp1a1* mRNA expression between experimental groups due to a low sample size (n = 3) and the use of brackish (11 ppt) rather than fresh water as a low salinity treatment. However, gill *atp1a1* mRNA expression within individuals is significantly correlated with rectal gland *atp1a1* mRNA expression (Pearson’s correlation coefficient R = −0.937, p = 0.006; Figure 3). This negative correlation, in which increased rectal gland *atp1a1* mRNA corresponds to decreased gill *atp1a1* mRNA, provides additional support for the hypothesis that the rectal gland and gill play opposite roles in elasmobranch osmoregulation with the latter potentially facilitating ion uptake at low salinities in addition to the more established role of the elasmobranch gill in acid–base regulation.

## Conclusions

Euryhaline elasmobranch species such as *D. sabina* can acclimate to a wide range of environmental salinities, and may be frequently challenged with significant salinity gradients over a short period of time. The ability of these taxa to maintain enantiostasis regardless of salinity relies upon the rapid and efficient balance of plasma osmolytes including sodium and chloride ions via tissues such as the gill, kidney, gut and rectal gland. Therefore elucidating the cellular and molecular mechanisms that facilitate ion balance provides critical insight into the physiology of euryhalinity, including the potential to understand why some taxa are successful across a wide range of salinity whereas others are not. This study represents a significant step in this direction, demonstrating gene regulation in the primary salt-secreting organ of a euryhaline elasmobranch following acclimation to a hyperosmotic environment. Future studies should further examine *atp1a1* gene transcription in the rectal gland and gill of *D. sabina* with regards to cellular mechanism e.g. promoter regulation, and also use *D. sabina* as a comparative model to examine the role and regulation of Na^+^/K^+^-ATPase in osmoregulatory tissues of euryhaline vs. stenohaline elasmobranch species.

## Abbreviations

*atp1a1*: Na^+^/K^+^-ATPase α1 subunit mRNA
*18S*: 18S ribosomal RNA
MS-222: tricaine methanesulfonate
qRT-PCR: quantitative reverse transcription-polymerase chain reaction
ppt: parts per thousand
